# Successful surgical management of a cerebellar subdural empyema in a domestic cat

**DOI:** 10.3389/fvets.2025.1655305

**Published:** 2025-09-15

**Authors:** Ian Hall, Martin Hamon, Aurelie Bruwier, Sara Michell González Blancas, Pierre P. Picavet

**Affiliations:** Department of Clinical Sciences, College of Veterinary Medicine, Kansas State University, Manhattan, KS, United States

**Keywords:** empyema, cerebellum, dura mater, craniotomy, otitis, cat

## Abstract

This case report describes the successful diagnosis and surgical management of a cerebellar subdural empyema in a 14-year-old Domestic Shorthair cat. The patient presented with a left-sided head tilt and right-sided hemiparesis. Magnetic resonance imaging (MRI) revealed a right-sided extra-axial cavitated lesion in the cerebellum, and mild right-sided otitis media without evidence of otitis interna. Surgical decompression was performed, guided by the use of a 3D-printed model, and purulent material was evacuated. Bacterial culture identified *Peptostreptococcus canis*, *Filifactor villosus,* and a Gram-negative rod population; all were suspectible to amoxicillin-clavulanic acid. Cerebellar subdural lesion histopathologic analysis confirmed a pyogranulamatous to lymphoplasmocytic inflammatory process. The patient showed rapid postoperative neurological improvement and was discharged with targeted antimicrobial therapy. Follow-up MRI at 2 months revealed resolution of the cerebellar lesion but progression of right-sided otitis media without evidence of otitis interna. A ventral bulla osteotomy was subsequently performed, and tympanic mucosa biopsy confirmed chronic inflammation with cholesterol granuloma formation. To our knowledge, this is the first report of successful surgical treatment of a cerebellar subdural empyema in a feline patient.

## Introduction

Focal intracranial infections are anatomically classified as brain abscesses (accumulations of purulent material within the CNS parenchyma), subdural empyemas (located between the dura mater and arachnoid mater), and epidural abscesses (situated between the dura mater and the inner surface of the skull) (1).

Intracranial bacterial infections in feline patients can occur via multiple routes, most commonly following bite wounds or as a complication of otitis interna (2). The heterogeneous neurological presentation is influenced by the size and anatomical location of the abscess or empyema, the mass effect exerted by the space-occupying lesion on adjacent neural tissue, and the inflammatory response elicited by the bacterial pathogens (2, 3).

Intracranial abscesses and empyemas are uncommon in feline patients, with most reported cases involving the cerebral hemispheres (2, 4). To date, only one case of a cerebellar empyema has been described, which resulted in euthanasia (2).

This report describes the first documented case of successful surgical management of a cerebellar subdural empyema in a feline patient, associated with an ipsilateral otitis media and without clinical or imaging evidence of otitis interna.

## Case presentation

A 14-year-old neutered male Domestic Shorthair cat was presented for evaluation of a 2-week history of ataxia and 5-day history of left-sided head tilt. The referring veterinarian had initiated treatment with prednisolone (0.5 mg/kg BID) which led to partial clinical improvement.

On presentation, vital signs were within normal limits. Neurological examination revealed left-sided head tilt and right-sided hemiparesis, with subjectively milder proprioceptive deficits in the right pelvic limb. Cranial nerve function was normal, and no spontaneous or positional nystagmus was detected. The combination of contralateral head tilt and ipsilateral postural reaction deficits was consistent with a paradoxical central vestibular syndrome, localizing the lesion to the right cerebellum, particularly the flocculonodular lobe or caudal cerebellar peduncle.

A complete blood count was unremarkable. Serum biochemistry revealed hyperglycemia (159 mg/dL; reference range: 70–130), mildly elevated creatinine (2.0 mg/dL; reference range: 0.9–1.9), and increased globulin concentration (5.4 g/dL; reference range: 2.6–4.5). Magnetic Resonance Imaging (MRI) of the head was performed under general anesthesia (Toshiba Vantage Galan 3 T scanner). Images were acquired before and after intravenous administration of Gadobutrol at a dose of 0.2 mg/kg.

**Figure 1:**
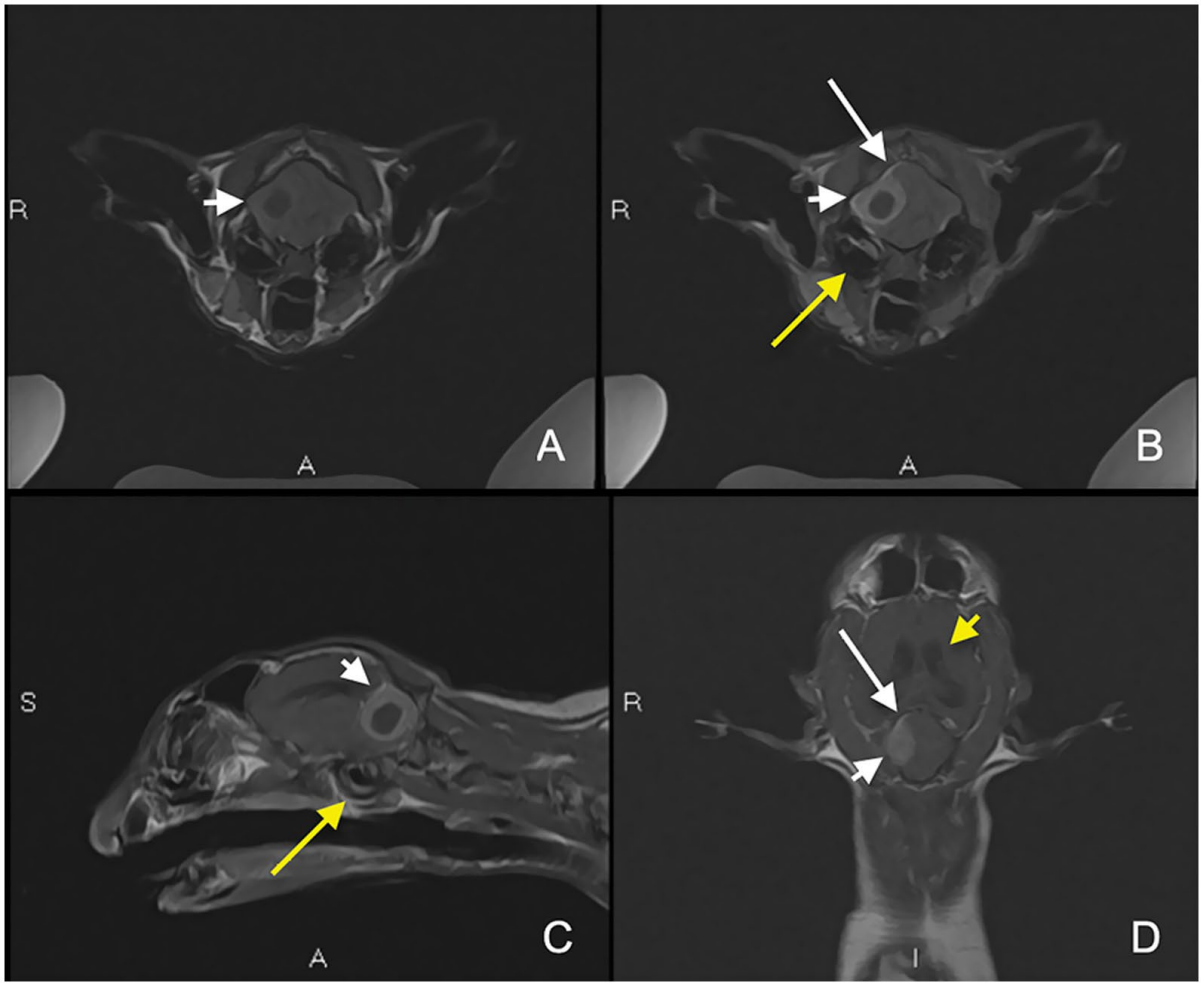
Magnetic resonance imaging (MRI) showing the right-sided extra-axial cavitated mass within the caudal fossa associated with a dural tail sign (long white arrow). Pre-contrast T1-weighted (T1W) axial **(A)**, post-contrast T1W fat saturated axial **(B)**, and post-contrast T1W sagittal **(C)** and dorsal **(D)** images showing a broad-based smoothly marginated mass with a T1W hypointense, non-enhancing central region surrounded by a large rim of strongly enhancing tissue (short white arrow). Mild otitis media (long yellow arrow) and ventriculomegaly (short yellow arrow) are also noted.

The MRI study revealed a right-sided extra-axial cavitated mass within the cerebellum, resulting in a significant mass effect, including caudal cerebellar herniation and obstructive hydrocephalus (Figure 1). The lesion was broad-based and smoothly marginated, occupying the right dorsolateral aspect of the caudal cranial fossa adjacent to the right occipital bone. It consisted of a centrally located, rounded, non-enhancing region that was hyperintense on T2-weighted (T2W) images, hypointense on T1-weighted (T1W) images, and demonstrated restricted diffusion. This core was surrounded by a rim of tissue that was isointense on T2W and strongly contrast-enhancing on T1W images, accompanied by focal meningeal enhancement (dural tail sign) along the rostral and dorsal margins. Marked compression of the adjacent cerebellar tissue was evident, along with moderate compression of the brainstem. Perilesional cerebellar edema was identified as T2W and T2-FLAIR hyperintensity within the surrounding cerebellar parenchyma. Additionally, there was a mild volume of gravity-dependent T2W hyperattenuating material within the right tympanic bulla, some of which is lining the luminal aspect of the bulla circumferentially with some enhancement. The differential diagnosis included both infectious and neoplastic etiologies. Imaging findings were consistent with purulent material within the cerebellum. An infectious process, particularly a subdural empyema of otogenic origin, is considered possible given the clinical presentation and the presence of concurrent otitis media. However, no significant abnormalities were detected in the inner ear or the intracranial structures at the level of the internal acoustic meatus. Other infectious considerations include a cerebellar abscess, fungal granuloma, or parasitic encephalitis, although these are less common. Neoplastic differentials include extra-axial tumors such as meningioma and lymphoma.

**Figure 2:**
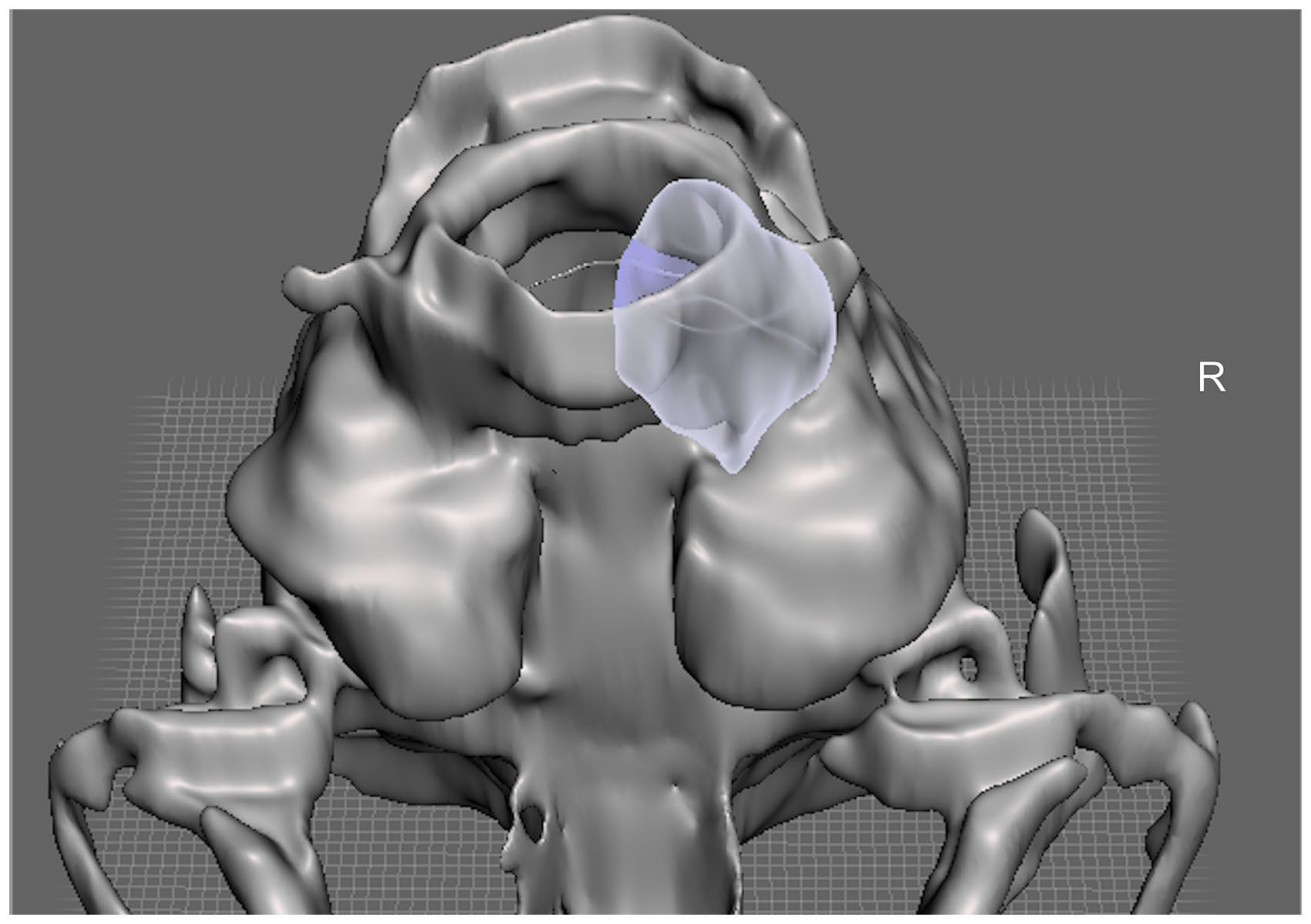
Illustration of the patient-specific 3D-printed modeling of the feline skull illustrating the targeted lesion highlighted in purple in a ventral view.

Prior to the procedure, a 3D-printed model of the skull was generated and utilized for detailed preoperative planning, allowing precise visualization of the anatomical structures and landmarks thus facilitating surgical approach. This model also served as an intraoperative reference to enhance spatial orientation during the surgery (Figure 2). The cat was positioned in sternal recumbency with the head elevated using a towel. A lateral approach to the right cerebellar fossa was performed in accordance with the technique described by Kent et al. (5). Following durotomy, careful dissection was carried out around the lesion. The tissue was noted to be friable and ruptured during manipulation, releasing purulent material. Samples were collected for anaerobic and aerobic bacterial cultures and histopathological analysis. The surgical site was thoroughly irrigated with sterile saline, and abnormal tissue was debrided. After extensive lavage, the dura mater was intentionally left open to facilitate postoperative drainage. The muscular and subcutaneous layers were closed using 3–0 polyglecaprone in a simple continuous pattern, and the skin was apposed with 4–0 nylon in a simple interrupted pattern. The cat recovered uneventfully from general anesthesia. Neurological examination performed the day following surgery demonstrated clinical improvement, with resolution of the head tilt and a reduction in the severity of the paresis. The patient was discharged 2 days postoperatively. At discharge, a 5-day course of prednisolone was prescribed (0.5 mg/kg orally every 24 h), along with amoxicillin-clavulanic acid for 60 days (20 mg/kg orally every 12 h). Buprenorphine was also prescribed for analgesia (20 μg/kg orally every 8 h transmucosally) for 7 days.

Histopathological analysis of cerebellar subdural lesion revealed a pyogranulomatous to lymphoplasmacytic inflammatory process. Anaerobic culture identified *Peptostreptococcus canis*, *Filifactor villosus*, and a predominant Gram-negative rod population. All isolated organisms were susceptible to amoxicillin-clavulanic acid. Aerobic culture showed no growth on original sample or enrichment.

**Figure 3:**
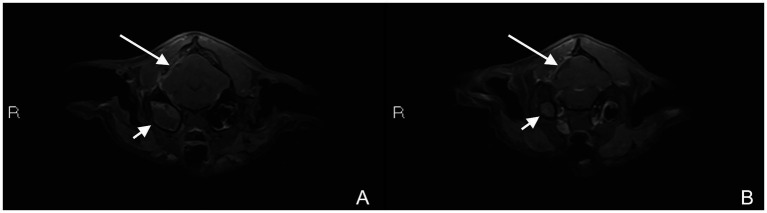
MRI study 2 months after surgery. Post-contrast T1W fat saturated axial images **(A,B)** showing postoperative changes associated with regional meningeal thickening and enhancement (long white arrow) and progressive right-sided otitis media (short white arrow).

Two weeks after surgery, the cat presented for routine suture removal. The neurological examination was completely normal at that time. The cat was rechecked 2 months after the decompressive surgery, while still on antibiotic therapy. The owners reported the cat to be completely normal, exhibiting normal behavior and activity levels with no observable neurological deficits. Physical and neurological examination were unremarkable. A follow-up brain MRI was performed 2 months postoperatively under general anesthesia, using the same protocol and dosage of intravenous Gadobutrol (0.2 mg/kg) for post-contrast imaging (Figure 3).

**Figure 4:**
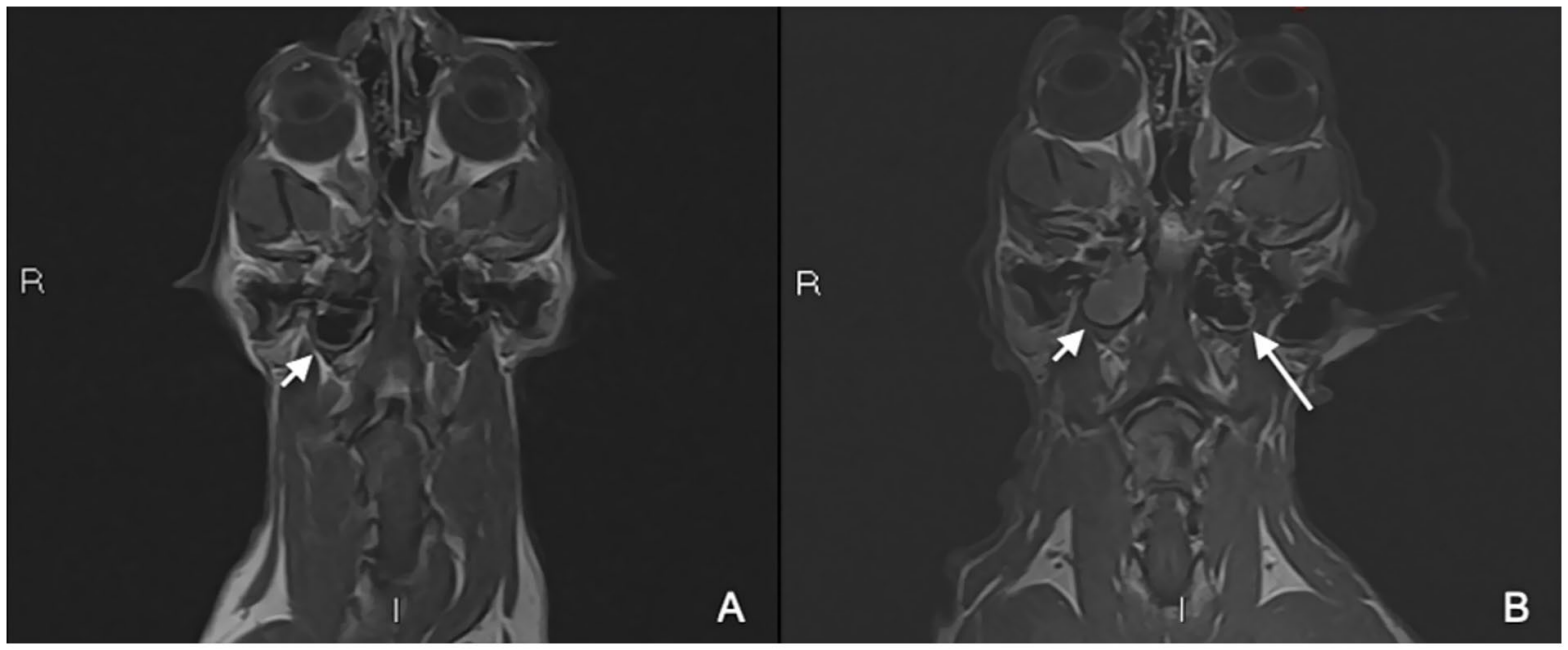
Pre-operative MRI study **(A)** and control MRI study 2 months after surgery **(B)**. Post-contrast T1W dorsal images **(A,B)** showing progressive right-sided otitis media (short white arrow) and suspected early left-sided otitis media (**B**, long white arrow).

Findings included mild diffuse contrast enhancement of the right temporalis muscle adjacent to the craniectomy site and a small wedge-shaped peripheral defect of the right cerebellar hemisphere with mild fluid accumulation isointense to cerebrospinal fluid. The T2W hyperintense material within the right tympanic bulla had increased in volume, filling both compartments and containing small intraluminal gas foci. In addition, new rim enhancement of the internal lining of the left tympanic bulla, most prominent dorsally, was identified. Those findings were in favor of focal right temporal myositis, regional meningeal thickening and enhancement suggestive of localized meningitis, and a small superficial cerebellar parenchymal defect with mild fluid accumulation isointense to cerebrospinal fluid, consistent with acquired hydrocephalus ex vacuo at the site of the previous extra-axial lesion, marked progression of right-sided otitis media (Figure 4) and early-stage left-sided otitis media.

Approximately 3 months after the initial neurosurgical procedure, a ventral bulla osteotomy (VBO) of the right tympanic bulla was offered and subsequently performed. Antimicrobial therapy was maintained until the time of surgery. Intraoperatively, mucopurulent material was present within the bulla. A sample was collected for bacterial culture, which yielded no microbial growth. Additionally, a biopsy of the tympanic mucosa was obtained, revealing chronic fibrosing mixed inflammation with cholesterol granuloma formation and mild to moderate multifocal glandular ectasia. The cat recovered uneventfully. An ipsilateral mild Horner syndrome was present postoperatively, characterized by miosis and mild protrusion of the nictitating membrane. The cat was discharged with 15 more days of amoxicillin-clavulanic acid (20 mg/kg orally every 12 h) and buprenorphine (20 μg/kg transmucosally every 8 h) for 7 days. At the telephone recheck 15 days after the surgery, clinical signs associated with the Horner syndrome had resolved and the cat was normal. The cat was rechecked 1 month after the right-sided VBO. Physical and neurological examination were unremarkable. At the telephone recheck 2 months after this procedure, owners reported that the cat was doing very well, had returned to a completely normal lifestyle, and declined further diagnostic investigations for the left-sided otitis media.

## Discussion

This case report describes the successful surgical management of a cerebellar subdural empyema in a cat. Bacterial infections of the CNS in cats are rarely reported and may arise via several routes, including direct extension from adjacent structures (e.g., otitis media/interna, nasal or orbital disease), penetrating trauma (such as bite wounds), or iatrogenic causes (2, 6). A previously reported case also described a subdural empyema secondary to hematogenous dissemination from a distant infectious focus (multifocal pneumonia) in association with mature neutrophilia (6). In contrast, no evidence of systemic infection or hematologic abnormalities was identified in our patient. This finding is consistent with previous studies (2, 4, 6, 7) and may indicate an absence of a systemic immune response to intracranial infection. Although mild ipsilateral otitis media was present on the initial MRI, characterized by a small volume of T2W hyperintense material partially filling the ventral compartment of the tympanic bulla without associated gas or marked mucosal thickening, there was no definitive evidence of concurrent otitis interna. Specifically, the MRI did not reveal abnormal signal intensity or contrast enhancement within the inner ear structures, bony changes of the petrous temporal bone, or direct communication between the middle/inner ear and the intracranial space (7–10). Consequently, an otogenic origin for the empyema was not immediately considered likely. Diagnostic myringotomy was not performed at the time of initial presentation for several reasons: the MRI demonstrated only mild middle-ear changes with minimal fluid accumulation, decreasing the likelihood of retrieving diagnostic material, and urgent neurosurgical decompression was prioritized to address the life-threatening mass effect. Moreover, culture of the purulent material obtained intraoperatively from the cerebellar lesion was considered more relevant for immediate antimicrobial selection. Definitive sampling of the middle ear was performed later during the VBO, once otitis media had clearly progressed on follow-up imaging. No extracranial or skull abnormalities were identified to suggest an alternative route of infection. Additionally, during dissection of the cranium intra-operatively, there was no visualization of a defect either in the dermis or cranium that would indicate a puncture wound causing bacterial introduction. No other diagnostic imaging was performed to assess a distant infectious focus. The significant progression of the right-sided otitis media observed over a two-month period on MRI strongly suggests that it was most likely the underlying cause of the subdural empyema, even though dissemination did not appear to occur through direct contiguous spread, as is usually the case. This progression occurred despite 2 months of targeted antibiotic therapy based on intraoperative culture results, which may reflect limited antimicrobial penetration into the middle ear, the presence of biofilm or residual inflammatory material, or chronic pathological changes that cannot be resolved by medical management alone. At the time of VBO, bacterial culture was negative, a finding that may be explained by prior prolonged antibiotic treatment, yet histopathology confirmed chronic inflammatory changes with cholesterol granuloma formation. Cholesterol granulomas have been associated with chronic otitis media in cats and have also been reported in a feline case of otitis media complicated by leptomeningitis (11). The development of left-sided otitis media may have resulted from ascending spread of subclinical nasopharyngeal infection, or undetected early-stage bilateral disease at initial imaging.

Certain intracranial tumors can exhibit MRI features overlapping with those of intracranial infections complicating their differentiation. Among the MRI characteristics suggestive of an abscess are a T2-weighted hypointense peripheral rim and/or susceptibility artifacts on T2*-weighted gradient echo sequences, attributable to paramagnetic substances generated during phagocytosis (12). These features, however, were not observed in the present case. Cystic meningiomas have been reported in both cats and dogs, representing approximately 6% of feline meningiomas and 13–32% of canine meningiomas (13, 14), with only two cases involving the cerebellum described in dogs (13). In human medicine, cystic meningiomas are classified into four subtypes, with Type I corresponding to centrally located intratumoral cysts—an imaging presentation closely resembling that of our case (15). Additionally, intratumoral abscesses within meningiomas have been documented in human literature (16, 17). The extra-axial lesion described here demonstrated multiple MRI features consistent with both cystic and abscessed meningiomas in human cases.

A retrospective analysis by Martin et al. (2) assessed 23 feline cases of intracranial empyema or abscessation, suggesting improved—but not statistically significant—survival following surgical treatment compared to medical management alone. To date, no consensus has been established regarding the optimal therapeutic approach in veterinary medicine. In human medicine, the management of intracranial infections typically involves a multimodal strategy combining surgical and medical interventions (18). Antibiotic monotherapy may be considered in select cases—specifically in patients with mild clinical signs, absence of significant neurological deficits, small purulent collections on imaging, and evidence of early clinical improvement—though close clinical and imaging surveillance is required. Prolonged antimicrobial therapy is often necessary to achieve resolution (18). In human medicine, otogenic intracranial empyemas are considered neurosurgical emergencies, and current guidelines recommend both urgent drainage of the intracranial collection and definitive surgical management of the primary middle-ear or mastoid source, often during the same anesthetic event if the patient is stable (19). In veterinary medicine, no formal guidelines exist for the management of intracranial infections secondary to otitis media/interna; however, a retrospective suggests that addressing both the intracranial lesion and the primary ear infection surgically may improve outcomes (9). In the present case, VBO was not performed at the initial craniectomy because the middle-ear changes were mild and of uncertain clinical relevance, and urgent decompression of the posterior fossa took priority. However, marked progression of otitis media on follow-up MRI despite prolonged antibiotic therapy warranted definitive surgical management, which was successfully achieved with VBO. VBO has been reported as a sole treatment for otogenic intracranial complications (7). However, given the progressive clinical deterioration, surgical decompression of the cerebellum was considered necessary.

Multiple surgical techniques have been described for accessing the caudal fossa in feline patients. Shores and Brisson (20) reported a large craniectomy extending from the foramen magnum rostrolaterally to the caudal parietal bone, which can provide improved visualization of ventrally located masses. However, this approach was not employed in the present case. Instead, the technique described by Kent et al. (5) was selected as it utilizes the visible landmarks of the tentorium ossium for surgical planning, thereby facilitating access to the cerebellar fossa while minimizing the risk of hemorrhage by preserving the transverse sinus vascular structures. Initiating the craniectomy caudal to the tentorium ossium attachment also reduces the risk of exposing the occipital lobe and limits soft tissue dissection. Given the dorsolateral location of the lesion in our patient, this approach was considered optimal. Additionally, 3D printing technology was used to convert advanced imaging data into a precise model of the feline cranium, enabling accurate visualization of the empyema’size, shape, and location. Klasen et al. (21) demonstrated the utility of 3D printing in surgical planning, highlighting its potential to decrease operative time and improve outcomes, particularly in oromaxillofacial procedures, though its applications extend broadly across surgical disciplines. In this case, the use of 3D printing was instrumental not only in guiding the surgical approach but also in optimizing preoperative planning and patient positioning.

Three distinct bacterial strains were isolated from the affected region: *Peptostreptococcus canis*, *Filifactor villosus*, and an abundant unidentified Gram-negative rod. CNS abscesses are often polymicrobial and more commonly associated with aerobic organisms—such as *Streptococcus*, *Staphylococcus*, *Pasteurella*, and *Nocardia* species—than with anaerobes like *Bacteroides*, *Fusobacterium*, *Peptostreptococcus*, *Actinomyces*, and *Eubacterium* species (4, 22–24). Both *P. canis* and *F. villosus* are Gram-positive, anaerobic bacteria within the Clostridial order and are recognized as part of the normal oral flora in dogs and cats (25, 26). Although the presence of oral-associated anaerobic bacteria suggested a potential bite wound as the source of infection, no external evidence of trauma was identified in this case. Interestingly, in cats, otitis media most often develops secondary to a nasopharyngeal polyp or an infection of the pharynx or upper respiratory tract that ascends through the Eustachian tube (27, 28). In this case, no nasopharyngeal polyp was evident on clinical examination or imaging, and there was no overt clinical history or signs consistent with active upper respiratory tract infection at presentation. However, cholesterol granulomas were found at the histopathologic examination within the middle ear, indicative of a chronic inflammatory process. Similar findings have been described in a postmortem study of a cat with otitis media and leptomeningitis caused by *Streptococcus canis* (11)*. P. canis* has already been reported in a cat with intracranial complications associated with otitis media/interna (7).

A VBO was performed to manage the otitis media, as imaging follow-up revealed worsening of the lesions despite 2 months of appropriate antibiotic therapy. Given the progression of the disease, first-line treatments such as prolonged antibiotic therapy or myringotomy were not considered appropriate (29). A VBO was elected as it is associated with a good prognosis in such cases, providing effective drainage and removal of infectious or inflammatory material from the tympanic cavity (7).

This case represents the first documented successful surgical treatment of a cerebellar subdural empyema in a cat, associated with isolated otitis media without evidence of otitis interna. It highlights the importance of considering otitis media as a potential source of intracranial infections in feline patients, even in the absence of otitis interna. Although no consensus exists regarding the optimal treatment, surgical intervention resulted in a rapid resolution of clinical signs.

## Data Availability

The raw data supporting the conclusions of this article will be made available by the authors, without undue reservation.

## References

[1] Bonfield CM Sharma J Dobson S. Pediatric intracranial abscesses. J Infect. (2015) 71:S42–6. doi: 10.1016/j.jinf.2015.04.012 25917804

[2] Martin S Drees R Szladovits B Beltran E. Comparison of medical and/or surgical management of 23 cats with intracranial empyema or abscessation. J Feline Med Surg. (2018) 21:566–74. doi: 10.1177/1098612X18792657 30106317 PMC10814531

[3] Lewis MJ Olby NJ Early PJ Mariani CL Muñana KR Seiler GS. Clinical and diagnostic imaging features of brain herniation in dogs and cats. J Vet Intern Med. (2016) 30:1672–80. doi: 10.1111/jvim.14526 27616749 PMC5032863

[4] Barrs VR Nicoll RG Churcher RK Beck JA Beatty JA. Intracranial empyema: literature review and two novel cases in cats. J Small Anim Pract. (2007) 48:449–54. doi: 10.1111/j.1748-5827.2006.00307.x 17543019

[5] Kent M Glass EN Schachar J. A lateral approach to the feline cerebellar fossa: case report and identification of an external landmark for the tentorium ossium. J Feline Med Surg. (2019) 22:358–65. doi: 10.1177/1098612X19869699 31418626 PMC10814664

[6] Cardy TJA Lam R Peters LM Malaren PJ Riera MM De Decker A. Successful medical management of a domestic longhair cat with subdural intracranial empyema and multifocal pneumonia. J Vet Emerg Crit Care. (2017) 27:238–42. doi: 10.1111/vec.12566 28079960

[7] Moore SA Bentley RT Carrera-Justiz S Foss KD Da Costa RC Cook LB. Clinical features and short-term outcome of presumptive intracranial complications associated with otitis media/interna: a multi-center retrospective study of 19 cats (2009–2017). J Feline Med Surg. (2019) 21:148–55. doi: 10.1177/1098612X18764582 29667535 PMC10814610

[8] Bancroft S Heinrich N Wolf C. Otogenic intracranial abscessation secondary to an inflammatory polyp with chronic otitis media/interna in a cat. Vet Rec Case Rep. (2022) 10:e499. doi: 10.1002/vrc2.499

[9] Sturges BK Dickinson PJ Kortz GD Berry WL Vernau KM Wisner ER. Clinical signs, magnetic resonance imaging features, and outcome after surgical and medical treatment of otogenic intracranial infection in 11 cats and 4 dogs. J Vet Intern Med. (2006) 20:648–56. doi: 10.1892/0891-6640(2006)20[648:csmrif]2.0.co;2 16734103

[10] Klopp LS Hathcock JT Sorjonen DC. Magnetic resonance imaging features of brain stem abscessation in two cats. Vet Radiol Ultrasound. (2000) 41:300–7. doi: 10.1111/j.1740-8261.2000.tb02077.x 10955490

[11] Van der Heyden S Butaye P Roels S. Cholesterol granuloma associated with otitis media and leptomeningitis in a cat due to a *Streptococcus canis* infection. Can Vet J. (2013) 54:72–3. 23814305 PMC3524819

[12] Carloni A Bernardini M Mattei C De Magistris AV Llabres-Diaz F Williams J. Can MRI differentiate between ring-enhancing gliomas and intra-axial abscesses? Vet Radiol Ultrasound. (2022) 63:563–72. doi: 10.1111/vru.13098 35509117

[13] May JL Garcia-Mora J Edwards M Rossemeisl JH. An illustrated scoping review of the magnetic resonance imaging characteristics of canine and feline brain tumors. Animals. (2024) 14:1044. doi: 10.3390/ani1471044 38612283 PMC11010916

[14] Kim K. Frontal cystic meningioma removed by a partial transfrontal craniotomy in a cat. Acta Sci Vet. (2021) 49:669. doi: 10.22456/1679-9216.112133

[15] Abdullah Alborihi SN Al-Awadi AA Abdullah Alborihi EN. A report of four cases of cystic meningiomas and a systematic review. Iran J Neurosurg. (2024) 10:E3. doi: 10.32598/irjns.10.3

[16] Sannareddy RR Lath R de Padua M Ranjan A. Meningioma with intra- and peritumoral abscess. Indian J Neurosurg. (2018) 7:220–2. doi: 10.1055/s-0037-1606820

[17] Patibandla M Addagada D Addagada G. Meningioma with intratumoral abscess: review of literature. J Surg. (2017) 2017:159. doi: 10.29011/2575-9760.000159

[18] Hall WA De Jesus O. Subdural empyema. In: StatPearls [internet]. Treasure Island, FL: StatPearls Publishing; (2024) Available online at: https://www.ncbi.nlm.nih.gov/books/NBK441858/32491761

[19] Penido Nde O Borin A Iha LC Suguri VM Onishi E Fukuda Y. Intracranial complications of otitis media: 15 years of experience in 33 patients. Otolaryngol Head Neck Surg. (2005) 132:37–42. doi: 10.1016/j.otohns.2004.08.007 15632907

[20] Shores A Brisson BA. Advanced technique in canine and feline neurosurgery: surgery of the caudal fossa. Hoboken, NJ: Wiley Blackwell (2023).

[21] Klasen JRS Thatcher GP Bleedorn JA Soukup JW. Virtual surgical planning and 3D printing: methodology and applications in veterinary oromaxillofacial surgery. Front Vet Sci. (2022) 9:971318. doi: 10.3389/fvets.2022.971318 36337192 PMC9635215

[22] Irwin PJ Parry BW. Streptococcal meningoencephalitis in a dog. J Am Anim Hosp Assoc. (1999) 35:417–22. doi: 10.5326/15473317-35-5-417 10493418

[23] Kent M. Bacterial infections of the central nervous system In: Greene CE, editor. Infectious diseases of the dog and cat. 3rd ed. St Louis: Saunders/Elsevier (2006). 962–74.

[24] Costanzo C Garosi LS Glass EN Rusbridge C Stalin CE Volk HA. Brain abscess in seven cats due to a bite wound: MRI findings, surgical management and outcome. J Feline Med Surg. (2011) 13:672–80. doi: 10.1016/j.jfms.2011.07.020 21872794 PMC7130018

[25] Harris S Croft J O’Flynn C Deusch O Colyer A Allsopp J. A pyrosequencing investigation of differences in the feline subgingival microbiota in health, gingivitis and mild periodontitis. PLoS One. (2015) 10:e0136986. doi: 10.1371/journal.pone.0136986 26605793 PMC4659563

[26] Reimer LC Sardà Carbasse J Koblitz J Ebeling C Podstawka A Overmann J. BacDive in 2022: the knowledge base for standardized bacterial and archaeal data. Nucleic Acids Res. (2022) 50:D741–6. doi: 10.1093/nar/gkab961 34718743 PMC8728306

[27] Shanaman M Seiler G Holt DE. Prevalence of clinical and subclinical middle ear disease in cats undergoing computed tomographic scans of the head. Vet Radiol Ultrasound. (2012) 53:76–9. doi: 10.1111/j.1740-8261.2011.01873.x 22092494

[28] Swales N Foster A Barnard N. Retrospective study of the presentation, diagnosis and management of 16 cats with otitis media not due to nasopharyngeal polyp. J Feline Med Surg. (2018) 20:1082–6. doi: 10.1177/1098612X17746282 29235932 PMC11104215

[29] Deleporte S Briand A Prelaud P. Clinical outcome of cats with suppurative otitis media and intact tympanum submitted to myringotomy: retrospective findings from 26 cases. J Feline Med Surg. (2024) 26. doi: 10.1177/1098612X241275286 PMC11450789 39344808

